# Examining Boards *vs.* Dental Colleges

**Published:** 1891-12

**Authors:** W. C. Barrett

**Affiliations:** Buffalo, N. Y.


THE
AMERICAN JOURNAL
—OF—
DENTAL SCIENCE.

VOL. XXV. BALTIMORE, DECEMBER, 1891. No. 8.

ARTICLE I.

### EXAMINING BOARDS *VS.* DENTAL COLLEGES.

BY DR. W. C. BARRETT, BUFFALO, N. Y.

It should be distinctly understood that the preparation of a paper upon this subject is not a self-imposed task, nor the title one of my devising. Your Business Committee expressly charged me with it, and I have never been one who shrank from doing my share at a dental meeting, even though the part assigned me were not that which would be most consonant with my feelings.

I now propose to speak my mind very freely, and if you do not like it, blame the Committee that urged me to the task, well knowing that I was not one who would mince matters. But in whatever I may say, let me in advance disclaim any thought of individual censure, or personal reflection. No one has been a more persistent critic of the schools than myself. I doubt if there be any man in dentistry who has so publicly and openly attacked them for their shortcomings. But when I saw that the things that I condemned were amended, that the colleges were really advancing as fast as professional opinion would permit, that the present system was fully up to that of other professional and literary schools, I gave them due credit for what they had accomplished. My strictures were prompted by an honest love for them and for my calling, by a desire to advance the cause of education, and not by the wish to level all our institutions of learning to the ground and groundlings, nor by any sense of prejudice or baseless jealousy.

Let me commence this paper by reviewing a little of the history of dental colleges. You all know how Harris was but coolly received when he proposed that the medical colleges should teach dentistry. And the medical schools were right in the refusal. The dentistry of that day was not worthy professional recognition. But Harris and his compeers knew only too well that there could be no profession without some system of professional education. The very term "Doctor" meant teacher, and if there were to be educated men there must be instruction and instructors.

The first dental college was thereupon founded in an experimental way. A new degree was inaugurated, one before unknown. To lift the great body of practicing dentists into an odor of respectability at once, without the tedious process of waiting for the ungraduated to die off in due process of nature, and to make popular the new distinction, it was conferred "Honoris Causa" upon a considerable number of the best exemplars of the dentistry of that day. The old Latin maxim of "Honores mutant mores" was kept in mind, and was in this instance proven true, for the new graduates *sine curriculo* became enthusiastic advocates of the fire-new degree and the shining fresh diplomas. The very charter of the college provided that any one might appear before its examining faculty, and if he was able successfully to pass the ordeal the degree of Doctor of Dental Surgery should be conferred upon him.

This step; so essential at the time, so fraught with final evil, accomplished its work. The end justified the means, and the degree became so popular that soon there was a call for more schools, and new institutions were founded, closely following the lead of the premier organization. The requirements for graduation were not very oppressive, nor the examinations too technical, for students were graduated at the end of one short term. The dogma that some very short sighted, unthinking men of to-day occasionally advocate, that "knowledge should be recognized wherever and however obtained," governed the conferring of degrees.

This principle, so specious in sound, so disastrous in consequences, soon produced its legitimate results. The faculties of the various institutions were the supreme judges as to qualification, and could establish any standard that they might choose. The requirements became less and less, until the culmination in the Delavan fraud in Wisconsin. The standard of that school was the name of dentist and the possession of the sum of twelve dollars. The "faculty" of that institution firmly believed in "recognizing knowledge wherever and however obtained." That was the very corner-stone upon which the school rested, and it did not essentially differ from the basis structure of other schools, except that it was more liberally constructed. Foreigners were graduated at most of the schools, without any knowledge of the English tongue in which the instruction was given. The colleges, you see, were "recognizing knowledge whenever and wherever obtained." The degree of Doctor of Dental Surgery fell into disrepute in foreign countries, because it was so promiscuously conferred. Foreign dentists of repute refused to fraternize with the graduates of American colleges. The very means that had been primarily employed to make the degree popular now operated to condemn it. The schools were killing their goose as fast as possible, by conferring honorary degrees in profusion, and by graduating incompetent men.

It was about this time that a few earnest men established a journal, that they might have an organ to express their very decided opinions upon these questions, and to labor for what they believed to be the best interests of the calling to which they had devoted their lives. They proposed to reform some things, or to batter out their own brains in trying. They could have no ulterior motives. They did not expect to make a cent, or to do much for themselves personally, save to gain a few enemies. The present speaker became the editor of that journal, and it is the one work of his life upon which he looks back with unalloyed satisfaction. We were a lot of Don Quixotes, with something more than a barber's bason in place of the helmet of Mambrino, and we were around looking for windmills to attack. The first one that had attracted our attention was this educational question. For one, I was certain that the ill-odor into which the D. D. S. was fast falling was due to the villainous system of "recognizing knowledge wherever and however obtained." It was a standard set upon a convenient sliding scale, and every school could interpret it to suit its own ideas. Under that plausible plea every irregularity that existed had found entrance. As long as that was the criterion, every school being its own judge of the quantity of knowledge to be recognized, the Delavan College was, in principle, as respectable as any other.

The *Independent Practitioner*, almost with the first number issued by the New York Dental Journal Association, in both its "Original Communications" and its "Editorial" departments, began very freely to discuss the subject. It asserted, what should be apparent to every intelligent mind, that colleges were not organized for the granting of degrees. Their business was to teach. They had no more right to "recognize knowledge whenever and wherever obtained" by the granting of a diploma upon a mere examination, than they had to pronounce upon the qualifications of clergymen or lawyers. Their sole duty was to conduct students through a prescribed curriculum of study, and this accomplished, it was proper to give them a certificate of the fact. The college diploma was just this certificate that a regular course of study had been pursued, and it could be no more. The schools could not, with any degree of propriety, attempt to pronounce upon the qualifications of any student, aside from the fact that he had finished a definite course of study.

Being fully satisfied of this, the *Independent Practitioner* began to attack the very foundations of the then method of conferring degrees. The journal bitterly opposed the acceptance of any term of practice as equivalent to a term of school. Twenty years of reputable practice had at first been accepted in lieu of a year's tuition. Then twelve years was deemed sufficient. This soon sunk to five years, and finally the most of the schools were practically graduating students upon the mere statement that the candidate called himself a respectable practitioner, and upon his nominal attendance upon one short course of lectures. The editor of the *Independent Practitioner* had for some time been accumulating evidence that some of our most reputable schools were making the attendance for the one winter a mere matter of form, and when he was properly loaded the fight began by the publication of an article attacking the oldest of our colleges for graduating some of the most reputable practitioners in America upon a mere examination. We thought that we might as well begin by aiming at the head. Nothing could be accomplished by charging upon those schools that had little of reputation anyway.

Advantage was taken of a clause in the Dental Act of the State of New York, which prescribes that a college must be recognized as reputable by the State Dental Society before its diplomas shall be accepted as a legal qualification. Charges were preferred against this same college for graduating students without attendance upon its full curriculum, and it was summoned to show cause why its name should not be stricken from the roll of reputable institutions. This was done, not in enmity to the college, but because a broad principle was involved, and the right or wrong of the matter could be best established by commencing at the top instead of the bottom.

The Dean appeared at the stated time before the Dental Society of the State of New York, and showed that the school had been but following an old and well-established practice, one that most of the schools accepted. It was with the utmost difficulty that some of those now within the sound of my voice were held in line, and prevented from quashing the whole proceedings by their votes. They did not fully comprehend the breadth of the attack that was made, and regarded it as the outbreak of a prejudice against our oldest dental college.

But the Dean frankly acknowledged that the college had become satisfied that the practice was an evil one, and they were but waiting until they could with propriety revoke the offer to so confer diplomas, and that the grand old school did not propose longer to postpone a step in advance. Upon this, the editor of the *Independent Practitioner* moved that the State Society express its entire confidence in the college, and pledge itself that it would very heartily support it in the new departure. From that time to this the Baltimore College of Dental Surgery has been foremost in the work of reform.

Then commenced the publication in the *Independent Practitioner* of a series of articles severely criticising and condemning all schools which granted diplomas without a faithful attendance upon the full term of study. The college which had been summoned before the New York State Dental Society, true to its enunciation of principles, took the initiative in organizing an association of the faculties of the best schools, and in raising the standard of graduation. It was discovered that all the colleges were sick and ashamed of the then lax condition, but that each was afraid to inaugurate a new system, because they had little confidence that the sentiment of the profession would sustain them, and feared that all the students would go to the institution which granted its so-called honors upon the easiest terms. Every enquiry from proposed matriculants was as to how quickly and with how little knowledge one could receive a diploma. It was the degree, and and not education, that men seemed to seek. But a solemn agreement was entered into by the leading schools, that diplomas should only be conferred in full course. The principle for which the *Independent Practitioner* had battled was conceded, and that journal at once announced that the fight was over. Henceforth it would be found a supporter of the schools, instead of their merciless critic, so long as that agreement was kept in good faith. The colleges having plainly evinced a desire to advance their own status, it was time to call off the dogs, and since then it has been mainly the puppies who have continued the war.

The compact entered into by the colleges has not only been respected by nearly all the schools, but the standard has been continually raised, just as fast as the sentiment of the dentists as a whole would permit. In fact, the Association of College Faculties is becoming a rather radical body. They have gone too fast, in the estimation of many. The term of pupilage has been extended, and the semesters have been lengthened. With this present term they open an obligatory course of three years instead of two, and of five months in place of three, while nearly or quite all of the best of them demand an attendance of seven months at each term. Already they are moving to make this nine, while a number of colleges have practically a school term of twelve months in each college year. At the last meeting of the Association of College Faculties, a resolution was introduced looking toward the extension of the term of pupilage to four years, instead of the three upon which they were just entering.

The graduates of fifteen years ago would now scarcely recognize their Alma Mater. The granting of honorary degrees has been practically abolished. No colleges can confer a diploma, except in course, without first obtaining the consent of all the other schools in the Association. Students must be in actual, daily, faithful attendance upon college lectures during the whole term of pupilage, or they are debarred from coming up for graduation. Any student who is twenty days behind in his attendance forfeits his privilege of examination. He must be as faithful in the laboratory and clinic rooms as in the class room. Every hour of every school day must be strictly accounted for. A daily roll call, at both lectures and clinics, is insisted upon. A graded course has been established, and the students divided into freshman, junior and senior classes. The curriculum has been greatly extended. More teachers have been added in all the schools, and wonderfully increased facilities provided.

The proportion of plucked or rejected men is constantly increasing. Entrance and passing examinations of a continually augmenting stringency are provided for. Every school is held to a strict accountability by its associate schools, for they keep a close watch upon each other. This responsibility of each college to all the rest, covers not only its manner of teaching and of granting diplomas, but its discipline and internal regulation as well. At the last meeting of the Associated Faculties one school was suspended from membership for a period of two years, because it had conferred a diploma upon a student who had not been faithful in a full attendance upon college lectures. This suspension is a serious matter, for it means that no college will receive a student from it or dismiss one to it, nor will its diplomas be recognized in any manner. The discipline must be effectual, unless the Examining Board of the State in which the school is located overrides the decision of the Association of College Faculties, and permits its graduates to enter upon practice, notwithstanding the irregularity. But if this is done there will be a grand explosion, that will blow somebody or something entirely out of the water.

All this has been accomplished in seven short years. Order has come out of chaos, and the complaint among those most concerned now is that the difficulties attendant upon graduation have been made too apparent, and that qualifications that are unreasonable are demanded. In fact, there is grumbling that it is now too difficult to obtain a diploma, especially upon the part of those old practitioners who are without any degree, and who claim that the schools should "recognize knowledge wherever and however obtained," and "place the proper stamp upon merit," they themselves having a rather clear idea as to where such knowledge and merit can be found.

The men who made the fight in the first place, and who may conscientiously claim to have had something to do with instigating the wonderful progress, are not now found among the howlers at the schools. They have watched the workings of the new order of things too closely not to be satisfied with the rate of advancement, and to recognize the conscientious efforts of the schools for a better state of things.

Who then are the critics of to-day? In the first place they are largely the ones who know least of the schools. They are too often dentists who do not keep up with the procession, and who do not know what has been achieved within the past seven years. They are in large part the echoes, the repeaters of the opinions of more original men. The majority of them are the men who shout with the biggest crowd, without exactly knowing what they are hallooing for. In the year 1884, there was a grand attack made upon the then methods of most of the schools, and a considerable number of the critics of to-day were their indignant defenders. In the course of five years they wakened from their lethargy, and to show their zeal and to exercise their voices they began to bark at the old hole, not realizing that the game was now in quite different quarters. In the course of five years more they will discover that they are on a false scent, and will then become the apologists for the schools, through thick and through thin.

We who were in the fight of 1884, well remember the clubs and stones that were thrown at us for disturbing the peace of Israel. American dentistry, we were told, was the pride of the world, and American dental schools were the *summum bonum* of all good. Those who criticised them were unpatriotic; were actuated by unworthy motives and bent upon the ruin of our educational system. But now, when a better condition has been inaugurated and the schools are redeeming their laurels, these men are the assailants. They are simply seven years behind the time—that is all.

The colleges are yet a long way from perfection. There is plenty of room for improvement. But the great reforms wrought within the past seven years, and the present continued rate of advancement, should bring immunity from the charges that they are conducted simply for pecuniary profit, and without a due regard for the best interests of dentistry. Indeed, it is not at all certain that dentists will sustain and support the colleges that are engaged in raising the standard. The schools outside the College Association are meeting encouragement in quarters where this shouid be least expected.

The organization of dental examining boards was but the natural outgrowth of the old methods of granting degrees. When the schools were graduating men upon a mere examination, and that too often but a pretense, there was an importunate call for some authority to scrutinize the work. Influential men in certain States secured the passage of laws authorizing the appointment of dental examiners, and in some instances these laws made such a board the supreme authority to regulate the practice of dentistry, utterly regardless of the only possible source of instruction. Then these men got themselves appointed members of such boards, and they had the neatest and tightest little system of irresponsible authority ever devised. So far as their own State was concerned, the profession was their vassal. Sometimes—by far too often—these Boards were composed of men who had never attended a dental school a day in their lives. But they were men of experience, and self-acquired knowledge. They knew nothing of the methods of the schools, but they knew what a dentist ought to be in daily life. They were ignorant of theory, but they knew practice. Their power was a wholesome check upon the schools.

The members of these boards naturally fell violently in love with their autocratic position as critics of the expert teachers, and as instructors and mentors of the pedagogues. They assumed an air of superiority and grasped the ferule threateningly. I am credibly informed that it was openly asserted in a meeting of the Association of Dental Examiners, and that too by a man whose diploma is only an honorary one, that they are the superior body and have the right to dictate to the colleges. Men who never passed an examination themselves assumed to examine others. Those who never were at school sat in judgment upon the methods of the schools.

Naturally this has caused irritation. The college faculties have doubted the ability of some of these men properly to conduct an examination of college graduates upon subjects with which the members of the board are not acquainted, and have asked that in case of the rejection of a graduate a list of the questions and answers should be furnished the school implicated. Of course, if the questions were fair ones and the candidate actually exhibited ignorance of that in which he should have been instructed, it would be a deadly blow to the college. One would have thought that this opportunity to smite the schools would not have been refused, but instead of the granting of the request a rather disdainful answer was returned, and the colleges were informed that they had no rights in the matter.

Thus a breach has been formed that is constantly widening, and that promises only evil to dentistry. There is a necessity for examining boards, and most certainly the colleges are essential. Let it not be forgotten that there is no other source of instruction. Examining boards do not teach. They only find fault with those who do teach. So far as education goes, they only sit in the breach and throw clubs. They give the students nothing, and if they but drive them away from the only source of instruction, I ask in what way are they a benefit to dentistry?

Let me not be understood as charging that this is a true picture of all the examining boards, or a faithful representation of every member. There are those who know our educational system very thoroughly, or knew what it once was, and they are imbued with nothing but the wish to serve the profession and the people. [We know that this is the case, for they have told us so themselves.] Will they not allow as much of disinterestedness in others? Are they the only ones actuated by worthy motives? While they are working faithfully in their chosen field, can they not allow the same privileges to their brethren? Some of them are broad minded and liberal in their views But a considerable proportion of the contemners of the schools are those who are least qualified to express an opinion.

The amount of the matter is, there is a great misunderstanding of the functions of a college. The schools cannot make dentists. They cannot impart brains, or gumption. They can do nothing but teach, and if a student is not disposed to learn they cannot do even that. Their business is simply to offer opportunities for obtaining an education. They can only lead the pupil through a specified curriculum of study, and when he has mastered that certify to the fact. If he has not the mechanical ability to make a pig-trough, they cannot impart it. They can teach him the theory of dentistry, and practical mechanics, but they cannot convey the ability to make a diagnosis.

There are all sorts of sodden fools in the pulpit. Do men blame the theological seminaries because every pastor is not an eloquent orator? There are stupid lawyers, who cannot properly conduct the simplest case in court. Is that the fault of their instructors? There are doctors who cannot distinguish a case of stomach-ache from a psoas abscess. Is that chargeable to the medical colleges? If it is because of professional ignorance the schools may be to blame, but if it is owing to mental incapacity the critic should go to the man's Maker, and not to his teachers.

But it may be answered that a student should not be accepted if he has not sufficient ability. What standard of talent shall be established, and who shall determine it? Should the common schools be clo ed to children who do not come up to some fanciful line of intelligence? The usual theory is, that the greater the lack of brains the greater the need for schooling. It is quite impossible for the dean of a college to determine who, out of some hundreds of proposed students, has all the ability requisite to make a first-class dentist. There is many a singed cat among the hayseeds who present themselves at a college door. The fellow with the hardest cheek and the best clothes is quite apt to be found at the foot of his class when the grand trial comes. The dean must receive a hundred men in two or three days. He does require that the applicant shall present evidence that he has a fair education—enough to enable him to comprehend technical studies—but mechanical and professional ability can only be determined upon an extended trial. Once having matriculated, the student has the right to pursue his course to the end, and if he can then demonstrate that he has been taught the things essential to know, he must receive the certificate to that effect.

All these things should be taken into consideration by the Boards of Dental Examiners, and they must try to work in harmony with the schools, or they will certainly go to the wall. Colleges are an absolute necessity. There can be no education without them. Examining boards have important functions, but let it not be forgotten that they can only live in the presence of the colleges, and that the sole reason for their existence is that they may assist the work of the schools, by insisting upon the thorough professional education that is provided only by the schools.

Our English brethren are meeting the same difficulties that we encounter. The students in their schools are not always the brightest minds, but I never heard that the teachers were blamed for that. A year ago I listened for two days to a debate in the British Dental Association, upon this very theme of education, and I found that Americans were not alone in their embarrassments. There is no Association of Colleges in England, and as a consequence there is no accepted standard, that of some schools being higher than that of others. So much is this the case, that graduates of those institutions whose requirements are the highest append the name of the school to their titles, an L. D. S. London, ranking higher than some others.

Had all the American Boards of Dental Examiners pursued the even tenor of their way, laboring as unostentatiously to advance the cause of dental education as has that of the State of New York, the present embarrassments would not have succeeded. But what is the consolation in getting upon the top of the fence unless one can shout and attract the attention of the passers by? Our Board has always confined itself to the discharge of its legitimate duties. With the single exception of our bastard State degree, one that has neither legitimate educational father or mother and which does not pretend to be an evidence of having pursued any course of study whatever, our law is a model of good sense, and our Board in the discharge of its duties may stand as an exemplar to the world. One does not hear from its members the invitation to look and see what great men they are, and how they are guiding the destinies of all Christendom. We hear no groan from them that, like Atlas, they support the world, but their influence is quite as potent in the State as if they magnified their office above all the earth.

If students who have gone through the full curriculum of any recognized college are to be examined by a State Board, it should be before their graduation. I have for some years advocated the appointment of a Board of Regents, before whom should appear all the students of every college in the State, and which alone should be empowered to grant degrees. This would place all the schools upon the same plane, and would give the proper encouragement to the well equipped, for students would seek the institution that gave the surest promise of graduation. It would take away all incentive to a shortening of the course, and would leave the faculties to the uninterrupted performance of their sole duty—that of teaching. Such a law has been passed by our Legislature, and is in force, or is about to be enforced, in the medical schools.

In the State of New York, as I have before said, no diploma is a sufficient qualification for practice until the school that granted it has been duly recognized by the State Society, acting through its Board of Censors. It is the duty of this board very carefully to enquire into the methods of teaching, the equipment and the general conduct of any school, and having determined that it is doing faithful, honest, legitimate work, it is placed upon the list of recognized schools, there to remain so long as it is worthy recognition. If at any time it prostitutes the power granted it, the society may, as it has done before, summon the school to answer any charges preferred against it, and if these are established it can be stricken from the list, and henceforth its diplomas are not worth so much as the paper upon which they are written, so far as this State goes.

That is a correct principle. The examining boards should keep an eye on the colleges, and hold them to a strict accountability. But to make no discrimination between the good and the bad, to condemn all alike and unheard, is to place a premium upon ignorance and dishonesty. It is to discourage education and to debase our calling to the level of a mere trade. It is the befouling of our own nest, which is said to be the characteristic of a dirty bird. No man will enter a college door when he is virtually informed by those to whom he looks for ready-made opinions that the diploma means nothing, that for his three years time and his hundreds of dollars expended he has nothing of value to show, and has not advanced himself in the estimation of those who should be the best qualified to judge.

No one who is fit for the position will become a teacher, if he is but to be the target for abuse and misrepresentation on the part of those who should stay his hands, and if he is to be charged by those who call themselves honorable men with being a cheat and a fraud. No, gentlemen. Our schools must be sustained if we are to maintain an honorable status among the callings of the world. Without schools and without a literature, dentistry will lose the honorable position it has gained, and you and I will sink to the level of the charlatan and the imposter.

---

BY PROFESSOR J. FOSTER FLAGG, PHILADELPHIA.

---

To my grateful appreciation of the invitation to attend the annual Union Meeting of the New York State District Dental Societies, I must add my sincere regret that I am unable to accept it. But I have felt that the mere title of the tenth essay, as given upon the official program, calls for, at least, the first of a proposed series of papers upon that range of subjects of which the discussion of "Examining Boards *versus* Dental Colleges" must form a most important part.

The cause of systematic dental education is one for for which I have labored, both earnestly and faithfully, for more than thirty years, and I have, therefore, had ample opportunity for observation as to the many advantages, as well as the numerous defects, which appertain to the developed plan which, while at present very properly, I think, partially accepted, is yet equally properly (I also think) very freely discussed, and, in large degree, very correctly censured.

It is for reasons too numerous to incorporate in this paper, but which are entirely conclusive in my mind, that I have hailed the formation of State Dental Examining Boards as a glorious possibility in the work of progressive dental education, and it therefore seems to me that to discuss these organizations at this day, after years of trial, as "Examining Boards *versus* Dental Colleges," is an astonishing illustration of the complete misapprehension as to the relations which should exist between colleges and boards.

I am too sadly aware of the shortcomings of college work, and even of such as were of necessity regarded as of the best, because, poor as they have been when viewed from the ideal standpoint, they have, nevertheless, actually been the best. I can truly say that I have striven unceasingly for the improvement of both means and opportunities for the gaining of knowledge, and for the most important work of its practical application, and yet I have recognized as fully as anyone how very much less than I have desired has been the result.

But on the other hand, I must assert for the credit of dental education as even now established, that we have sufficient evidence that much more competent young practitioners, in larger proportion, are prepared for practice than have ever been produced by any previous method. I have also had quite as much knowledge of the "human frailty" side of faculty work as has any outsider.

I know that candidates for graduation can answer questions asked by their teachers much better than when put in a different way by other examiners, and though I would readily admit that a thorough knowledge would preclude this possibility, I would yet ask, who among all of us could gain that thoroughness of knowledge appertaining to the various subjects comprising the education of a dentist, in a three year's course, or a ten years' course, or even in anything less than a lifetime of study and practice?

I know that knowledge of hard work, constant attendance, excellent deportment and evident love for the work and earnest desire to learn, upon the part of the candidate, will so soften the hearts of members of faculties that they will vote upon the "reasonable hope for the future," rather than upon the absolute attainments of the present; and I ask, who among you can say that he would not do the same?

I know that all kinds of deception can be practiced by unprincipled, short-sighted candidates, and that it is practically impossible that faculties should not be thus deceived, though it is taught, most warningly, that those who act thus deceive themselves the most of all—but even then the temptation is too great, so long as a diploma continues to be a guarantee of right to practice.

And with this knowledge, do you wonder that I have hailed with joy the great possibilities, as co-workers in the cause of education, of the Dental Examining Boards?

And do you wonder at my astonishment at, and my protest against, the title "Examining Boards *versus* Dental Colleges"? Instead of *versus*, it should be *paritas* or *simul*, always!

What general would organize an army in such wise that in battle his artillery would be *versus* his infantry? What kind of city government would that be with its police *versus* its mayor and councils?

And so, if in any work the departments should be shoulder to shoulder, united by the strongest bonds, ignoring self-interest for the love of the greater work (if that be humanly possible), this should exist in education.

It is upon education that the highest hopes of the human race are based. It is the perfecting, or at least the improving, of educational work, which is the grandest problem of our day. It is a task which is truly Herculean, and which therefore demands the most conscientious endeavor and the most united effort.

That good may be the outcome of this work is my sincere desire, and it is to this end that I give you as my "sentiment" "Examining Boards AND Dental Colleges."

---

BY PROFESSOR C. N. PIERCE, PHILADELPHIA.

---

* * * I should very much enjoy being with you at your coming meeting, and the giving of my views on education, but they cannot be considered as from a dental college standpoint, because I am alone of all the teachers, and have been much criticised by the faculties of other schools.

But, notwithstanding this, I hold, as I have done for several years, that the hope of our profession and of our best schools is in the "State Boards of Dental Examiners." We cannot have the standard desired until the power of granting degrees is beyond the control of teaching faculties, and vested iu the hands of a *competent* board. I know that the excuse is made that boards as now organized are oftentimes the subjects of political intrigue or favoritism, but we must recollect that they are yet new, and are now passing through an evolutionary process, and it will take some time to have what is desired or required; but it will come in due time.

But with all these imperfections, they are safer to-day to judge of the qualifications of the candidate for practice, than a faculty depending wholly upon the class for support or compensations for exhausting labor rendered in teaching.

---

BY PROF. FRANK ABBOTT, NEW YORK.

---

* * * It would afford me much pleasure to accept your invitation to be present at your meeting, and if I were able to leave my post I should certainly be there. But I do not attach as much importance to the discussion of the subject,

"Examining Boards *versus* Dental Colleges," as you do. In the first place, there is no such question before the profession, that I am aware of. The examining boards and college faculties are not working or talking against each other; consequently any discussion of such a subject, in my opinion, would be altogether out of place.—*Dental Practitioner and Advertiser.*

